# Fine-Tuning the PI3K/Akt Signaling Pathway Intensity by Sex and Genotype-Load: Sex-Dependent Homozygotic Threshold for Somatic Growth but Feminization of Anxious Phenotype in Middle-Aged PDK1 K465E Knock-In and Heterozygous Mice

**DOI:** 10.3390/biomedicines9070747

**Published:** 2021-06-28

**Authors:** Mikel Santana-Santana, José-Ramón Bayascas, Lydia Giménez-Llort

**Affiliations:** 1 Institut de Neurociències, Universitat Autònoma de Barcelona, 08016 Barcelona, Spain; mikel.santana@e-campus.uab.cat; 2 Department of Psychiatry and Forensic Medicine, School of Medicine, Universitat Autònoma de Barcelona, 08016 Barcelona, Spain; 3 Department of Biochemistry and Molecular Biology, School of Medicine, Universitat Autònoma de Barcelona, 08016 Barcelona, Spain

**Keywords:** RDoC, PI3K/Akt, signaling pathway, sex, genetic load, fine tuning, anxiety, aging

## Abstract

According to the Research Domain Criteria (RDoC), phenotypic differences among disorders may be explained by variations in the nature and degree of neural circuitry disruptions and/or dysfunctions modulated by several biological and environmental factors. We recently demonstrated the in vivo behavioral translation of tweaking the PI3K/Akt signaling, an essential pathway for regulating cellular processes and physiology, and its modulation through aging. Here we describe, for the first time, the in vivo behavioral impact of the sex and genetic-load tweaking this pathway. The anxiety-like phenotypes of 61 mature (11–14-month-old) male and female PDK1 K465E knock-in, heterozygous, and WT mice were studied. Forced (open-field) anxiogenic environmental conditions were sensitive to detect sex and genetic-load differences at middle age. Despite similar neophobia and horizontal activity among the six groups, females exhibited faster ethograms than males, with increased thigmotaxis, increased wall and bizarre rearing. Genotype-load unveiled increased anxiety in males, resembling female performances. The performance of mutants in naturalistic conditions (marble test) was normal. Homozygotic-load was needed for reduced somatic growth only in males. Factor interactions indicated the complex interplay in the elicitation of different negative valence system’s items and the fine-tuning of PI3K/Akt signaling pathway intensity by genotype-load and sex.

## 1. Introduction

The expression of psychiatric symptoms such as anxiety across lifespan still needs important research efforts to dissect and understand their modulation’s biological and environmental basis [1]. Here, the new understanding of psychopathology in terms of dysregulation and dysfunction in essential behavioral features through neurobiology and behavioral neuroscience can provide a promising research scenario [2,3]. In this new conceptualization, fear, aggression, and distress are three draft constructs within the negative valence system (NVS), one of the five domains in the NIMH’s Research Domain Criteria (RDoC) matrix [2,4]. This RDoC matrix comprises the interplay between behavioral dimensions or functional constructs inspected by seven different ‘units of analysis’, namely, genetic and molecular basis, cells and neuronal circuits, physiology of the phenotypes, behavior, and self-report [3]. The molecular genetic basis of NVS phenotypes is considered to be in its infancy, yet with few candidate genes nominated for anxiety disorders [5]. In this context, basic research on signal transduction provides an advanced close examination of the impact of cell membrane receptors and second messengers on cellular biochemistry and physiology. However, the downstream actions unveiling how these genetic aspects translate into anxiety-related NVS constructs are challenging to characterize, mainly when related to biological factors as sex or the genetic load in aging scenarios, since female mice are underrepresented in research [6] and aging animals are scarcely studied [7].

Different PDK1/Akt mutant mice have consistently manifested a higher depressive and/or anxiety-like behavior [8,9,10,11]. The 3-phosphoinositide-dependent protein kinase-1 (PDK1) [12], an enzyme that activates Akt among other AGC kinases [13,14], is a key transmitter of extracellular signals of the phosphatidylinositol 3-kinase (PI3K) signaling, a pathway extensively involved in controlling neuronal development and function [15]. Its functional role in cellular biochemistry and physiology, as well as cancer and metabolism, has been largely explored [16], although in recent years, further evidence has been generated about its role in bipolar disorder, depression, anxiety, and schizophrenia [17,18,19,20,21,22,23]. In addition, antidepressants, antipsychotics, and mood stabilizers modify Aktactivity [17,24,25,26]. Moreover, the PDK1/Akt pathway has been also linked with suicide, alcohol drinking, and post-traumatic stress disorder [27,28,29]. Recently, a non-pharmacological intervention attenuated the cognitive deficit and depressive/anxiety-like behaviors induced by a stressor through the recovery of hippocampal Akt activity [30]. In addition, after postnatal maternal separation, mice treated with an early life non-pharmacological intervention showed ameliorated depressive and anxiety-like behavior through enhanced phosphorylated Akt in the hippocampus [31].

Here, mutant mice for this signaling pathway provide an experimental tool to depict the nuances of its downstream modulation. Mutation of PDK1 Lys465, a residue forms key interactions with the D3 and D4 phosphates of the PtdIns(3,4,5)P3 second messenger, to a Glu residue abolished binding of PDK1 to phosphoinositides and localization at the plasma membrane [32]. This signaling lesion selectively affected the phosphorylation and activation of PKB/Akt isoforms, but left intact the activation of other AGC kinase-family members [33]. These mice present smaller body size and insulin resistance [33]. At the brain level, they also exhibit pronounced Akt signaling deficits in both the cortex and the hippocampus during young adulthood (3–4 months of age) but tend to be attenuated by middle age (11–14 months of age) [34,35]. We recently showed that the double mutation of the PDK1 PH-domain (PDK1−/−) resulted in an enhancement of NVS shown as an increase of responses of fear and anxiety-like behaviors in anxiogenic situations [36]. Interestingly, this seemed to be specific to young adulthood and was found regulated at middle age. In contrast, as measured in a spatial working memory task, cognitive deficits were found in both young and mature mutants and independently of the level of their anxious-like profiles. These distinct age- and function-dependent impacts would agree with the distinct cortical and limbic deficits in the Akt signaling in their brains [34]. The elicitation of age- and regional-dependent specific patterns suggests that fine-tuning the PKB/Akt signal intensity that enables diverse physiological responses also has in vivo translation into the NVS, and age is a key regulatory factor.

Although women are significantly more likely than men to develop an anxiety disorder throughout the lifespan [37], fewer than 45% of animal studies into mood disorders used females [38,39]. The contribution of sex and genetic load in the anxious-like behavioral phenotype in this particular animal model is still unknown. Our previous report suggested that sex differences should be further explored [36]. Regarding genetic load, we hypothesize that heterozygous mice (PDK1+/−) may differ behaviorally from wild-type mice and/or homozygous mice due to differences in the intensity of the Akt signal under physiological conditions. On the other hand, recently, Akt deficiency in Akt isoform mutant mice altered anxiety-like behavior in an isoform- and sex-specific manner [40]. However, those sex-specific behavioral differences could not be explained by Akt expression or activation differences between the sexes.

Therefore, the present study aimed to explore further the contribution of sex and genetic load in the expression of the somatic and anxious-like behavioral phenotype of the PDK1-K465E PH-domain knock-in mice.

## 2. Materials and Methods

### 2.1. Generation of PDK1^K465E/K465E^ Mice and Genotyping Analysis

The generation and genotyping of the PDK1 K465E/K465E knock-in mice expressing the single-amino-acid substitution of lysine 465 to glutamic acid in the PDK1 PH domain were described previously [17]. The mice were subjected to PCR genotyping of genomic DNA isolated from ear biopsy using primers K465E F (5′-GGG TGA AGC ATG GAA TCT GTG TCT T) and K465E R (5′-GCC AGG ATA CCT AAG AGT ACC TAG AA). PCR amplification resulted in a 196-bp product from the wild-type allele and a 236-bp product from the targeted allele.

### 2.2. Animals

A total of 61 mature age (MA, 11–14-month-old) mice, PDK1^−/−^ (14 males, 16 females), PDK1^+/−^ (8 males, 10 females) and PDK1^+/+^ (also referred to as WT, 6 males, 7 females) were used.

Mice were maintained at the Animal House Facility of the Universitat de Lleida under standard husbandry conditions (housed three to four per cage in Macrolon cages, 35 × 35 × 25 cm, with food and water ad libitum, 22 ± 2 °C, a 12 h light: dark cycle and relative humidity 50–60%). Behavioral assessments and data analysis were performed blind to the experiment, in a counterbalanced manner, in the light cycle, from 9:00 to 13:00 h. All procedures were in accordance with Spanish legislation on ‘Protection of Animals Used for Experimental and Other Scientific Purposes’ and the EU Directive (2010/63/UE) on this subject. The study complies with the ARRIVE guidelines developed by the NC3Rs and aims to reduce the number of animals used [41].

### 2.3. Behavioral Assessments

**Figure 1:**
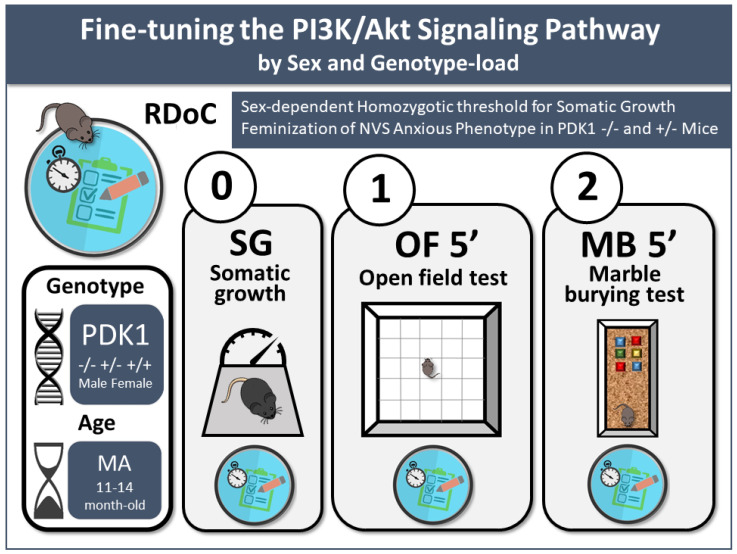
Graphical abstract. Fine-tuning the PI3K/Akt signaling pathway by sex and genotype-load. Experimental design: 3-days battery to assess somatic growth and the Research Domain Criteria (RDoC) negative valence system (NVS) in male and female PDK1−/−, PDK1+/− and PDK1+/+ mice at mature age (MA) are illustrated. Main findings are also indicated.

Animals were behaviorally assessed for NVS in the open field [42] and the marble-burying tests [43], two unconditioned tests differing in their anxiogenic conditions. A graphical abstract, also including the conclusions, illustrates the methodological setting and procedures (Figure 1).

As measured by body weight, somatic growth was recorded on Day 0 prior to the behavioral battery of tests to monitor possible confounding factors. Lack of sensorimotor problems was already described in these animals in the precedent work [36].

Day 0. Somatic Growth/Bodyweight. Bodyweight was used to measure the somatic growth and physical condition/health status of animals.

Day 1. Open field test (OF). Animals were individually placed in the center of an illuminated (20 lux) open field (homemade woodwork, white box, 55 cm × 55 cm × 25 cm) and observed for 5 min. First, the ethogram of action programs (sequence of behavioral events) was analyzed. Thus, the duration of freezing behavior (latM, latency of movement) and the latency of the behavioral events that follow it were recorded: leaving the central square (latC), reaching the periphery (thigmotaxis) (latP) and performing first wall rearing (latR). Second, the time course and total levels of exploratory activity were measured as horizontal (C, number of crossings) and vertical (Rw, rearing with wall support) locomotor activity. Finally, as previously described [44] we evaluated the presence of bizarre behaviors assessed through the number of stereotyped rearings without wall support (Rc).

Day 2. Marble-Burying Test (MB). The procedure used was as previously described [45]. The mice were placed individually facing the wall in a standard home cage with six glass marbles (1 cm × 1 cm × 1 cm) on a 5-cm-thick layer of clean wood cuttings. The marbles were spaced in three rows of two marbles per row in one half of the cage. The mice were left in the cage with marbles for a 30-min period. The test was terminated by removing the mice. The number of marbles that were buried, changed position (partially buried or turned), and were left intact (I) were measured.

### 2.4. Statistics

Statistical analyses were performed using SPSS 15.0 software. All data are presented as mean ± SEM, and are illustrated as bars that illustrate the mean values in each group segregated by genotype and/or sex, as indicated in the legends. To evaluate the effects of the genotype (G) and sex (S) group, a 3 × 2 factorial analysis design was applied. Differences were studied through Multivariate General Lineal model analysis, followed by post hoc Sidak test comparisons when it was possible. For categorical variables, the Fisher’s exact test was used. Graphics were made with GraphPad Prims 6, and *p*-value < 0.05 was considered statistically significant.

## 3. Results

### 3.1. Somatic Growth/Bodyweight

**Figure 2:**
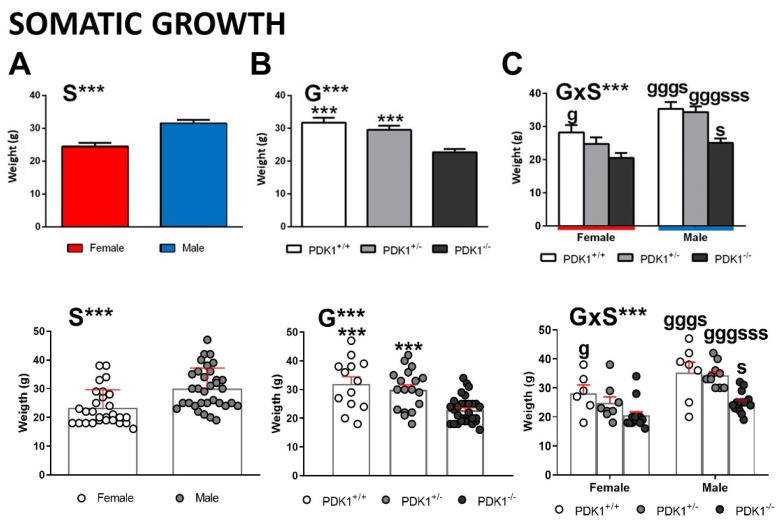
Somatic growth/ body weight in female and male mature PDK1^−/−^, heterozygous PDK1^+/−^ and homozygous WT (PDK1^+/+^) mice. Top panel: Data are expressed as mean ± SEM. Bottom panel: Individual data are depicted. Bars illustrate the genotype or sex groups. Factorial analysis: (**A**) S, sex effect; (**B**) G, genotype effect; (**C**) G × S, genotype × sex interaction effects; *** *p* < 0.001. *Post-hoc* test: genotype: ^g^ *p* < 0.05, ^ggg^ *p* < 0.01 vs. the corresponding KO (PDK1^−/−^, black bar) group; s (sex), ^s^ *p* < 0.05, ^sss^ *p* < 0.001 vs. the corresponding male of the same genotype.

The bodyweight of animals showed sex, genotype, and interaction effects. A part of sexual dimorphism (Figure 2A, S, F_(1,55)_ = 22.035, *p* = 0.000), significantly lower body weight of PDK1^−/−^ mice than WT and heterozygous mice was observed (Figure 2B, G, F_(1,55)_ = 15.353; *p* = 0.000; PDK1^+/+^, ***, *p* = 0.000; PDK1^+/−^, ***, *p* = 0.001). When analyzed per sex and genotype (Figure 2C, G × S, F_(1,55)_ = 22.035, *p* = 0.000), post hoc comparisons showed that the higher body weight in males is preserved in all the genotypes (Figure 2C, PDK1^+/+^, s, *p* = 0.024; PDK1^+/−^, sss, *p* = 0.001; PDK1^−/−^, s, *p* = 0.030). Moreover, in females, somatic growth followed a progressive decrease with genotype load (Figure 2C, PDK1^+/+^, g, *p* = 0.018), but heterozygous males had normal weight (Figure 2C, PDK1^+/+^, ggg, *p* = 0.000; PDK1^+/−^, ggg, *p* = 0.000).

### 3.2. Open Field Test

**Figure 3:**
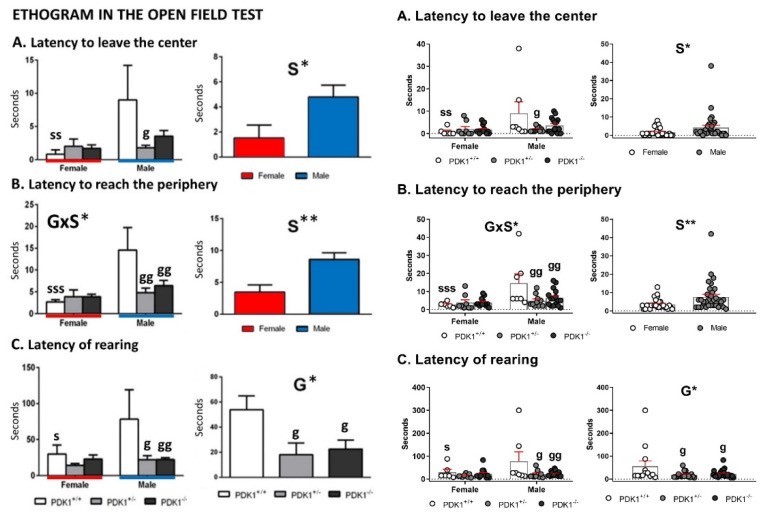
Ethogram in the open-field test in female and male mature PDK1^−/−^, heterozygous PDK1^+/−^ and homozygous WT (PDK1^+/+^) mice. ((**A**–**C**) **Left panel**): Data are expressed as mean ± SEM or incidence. Bars illustrate the genotype groups. ((**A**–**C**) **Right panel**): Individual data are depicted. Factorial analysis: G, genotype effect; S, sex effect, * *p* < 0.05, ** *p* < 0.01. *Post-hoc* test: genotype: ^g^ *p* < 0.05, ^gg^ *p* < 0.01 vs. the corresponding WT (PDK1^+/+^) group; s (sex), ^s^ *p* < 0.05, ^ss^ *p* < 0.01, ^sss^ *p* < 0.001 vs. the corresponding male of the same genotype.

**Figure 4:**
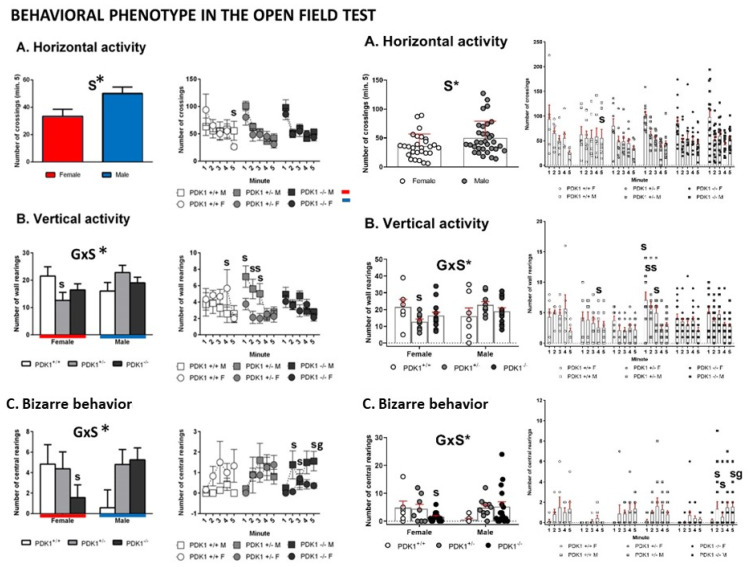
Exploratory and bizarre behaviors in the open-field test in female and male mature PDK1^−/−^, heterozygous PDK1^+/−^ and homozygous WT (PDK1^+/+^) mice. ((**A**–**C**) **Left panel**): Data are expressed as mean ± SEM or incidence. ((**A**–**C**) **Right panel**): Individual data are depicted. Bars illustrate the genotype groups, as indicated in the Y-axis. Symbols illustrate the different groups (left panel) or individual values (right panel), as depicted in the legends or abscissae. Factorial analysis: G, genotype effect; S, sex effect, * *p* < 0.05. Post hoc test: g (genotype), ^g^ *p* < 0.05 vs. the corresponding WT (PDK1^+/+^) group; s (sex), ^s^ *p* < 0.05, ^ss^ *p* < 0.01 vs. the corresponding male of the same genotype.

Figure 3 and Figure 4 depict the main behavioral domains, events, and units of analysis in the open-field test, showing the distinct sex-dependent performances of homozygous and heterozygous PDK1 mutants compared to WT groups.

Fear and Thigmotaxis—Immediate response to exposure to the open-field was similar among groups, with no differences in the latency of first movement (not shown). Sex differences were found in the latency to leave the center (Figure 3A, S, F_(1,55)_ = 5.538; *p* = 0.022) and to reach the periphery (Figure 3B, S, F_(1,55)_ = 11.057; *p* = 0.002) with females being faster than males. Post hoc multicomparison analysis showed that this difference was due to sex dimorphism in the behavior of WT mice (Figure 3A, lat center, ss, *p* = 0.006), since PDK+/− and PDK1−/− males also left the center faster than WT males, albeit this difference only reached the statistical significance in the heterozygous group (Figure 3A, lat center, g, *p* = 0.018). This genotype × sex interaction reached statistical significance in the latency to reach the periphery (Figure 3B, G × S, F_(2,55)_ = 4.028; *p* = 0.023). Post hoc multicomparison analysis showed that WT females arrived faster than males (Figure 3B, lat periphery, sss, *p* = 0.000) and that both PDK1+/− and PDK1−/− males also reached the periphery sooner than WT (Figure 3B, gg, *p* = 0.003 and gg, *p* = 0.008, respectively).

Vertical behavior—Latency of rearing showed a genotype main effect (Figure 3C, G, F_(1,55)_ = 3.675; *p* = 0.032), where both PDK1+/− and PDK1 −/− genotypes performed rearing earlier than WT (Figure 3C, *, *p* = 0.035 and *, *p* = 0.040, respectively). Post hoc multicomparison analysis also showed that latency of rearing was shorter in female WT as compared to males (Figure 3C, s, *p* = 0.031) and that both PDK1+/− and PDK1−/− males performed rearing earlier than WT males (Figure 3C, g, *p* = 0.016 and gg, *p* = 0.008, respectively). As shown in Figure 4, the min-by-min analysis of the temporal course of horizontal locomotor activity indicated similar habituation curves in the three genotypes. In the last minute of the test, the female sex performed less activity than males (Figure 4A, S, F_(1,55)_ = 5.763; *p* = 0.020). Post hoc multiple comparison analysis indicated that this sex effect was mostly due to the sexual dimorphism of WT mice in minute 5 (Figure 4A, s, *p* = 0.043).

Vertical activity (Wall Rearing): No main but interaction effects were found (Figure 4B, G × S, F_(2,55)_ = 3.340; *p* = 0.043). Post hoc multiple comparison analysis showed differences in the heterozygotes where males outperformed more than heterozygote females in minutes 1 (s, *p* = 0.036), 2 (ss, *p* = 0.006), and 3 (s, *p* = 0.025), resulting in a total higher total vertical activity (Figure 4B, s, F_(1,55)_ = 6.631, *p* = 0.013). WT females showed a higher rearing behavior in minute 4 (s, *p* = 0.047).

Bizarre behavior (Rearings in the center): No main but interaction effects were also found in the rearings performed in the center of the apparatus (Figure 4C, G × S, F_(2,55)_ = 3.360; *p* = 0.043). Post hoc multiple comparison analysis showed homozygote mutant females performed less than homozygote mutant males in minutes 2 (s, *p* = 0.034), 3 (s, *p* = 0.048), 5 (s, *p* = 0.018), resulting in a total lower vertical rearing activity in the center (Figure 4C, s, F_(1,55)_ = 4.700, *p* = 0.035). Moreover, in minute 5, homozygote mutant males exhibited more than wild-type mice (Figure 4C, g, *p* = 0.039).

### 3.3. Marble Burying Test

**Figure 5:**
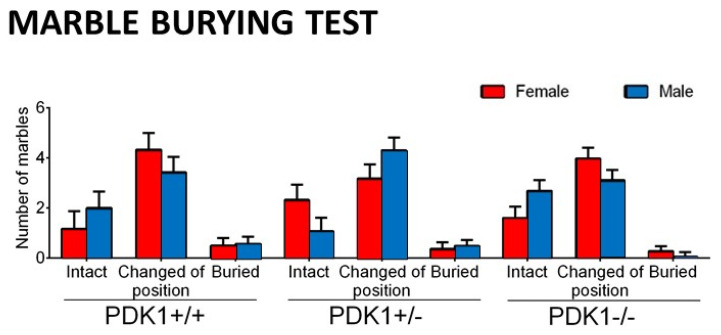
Marble burying test in female and male mature PDK1^−/−^, heterozygous PDK1^+/−^ and homozygous WT (PDK1^+/+^) mice. Top panel: Data are expressed as mean ± SEM or incidence. Bottom panel: Individual data are depicted. Bars illustrate the genotype groups, as indicated in the Y-axis. Symbols illustrate the different groups or individual values, as depicted in the legends or abscissae. Factorial analysis: G, genotype effect; S, sex effect; all *p* > 0.05, *n.s*.

The qualitative (three levels of interaction) and quantitative (number) analysis of the marble-burying test did not show any statistically significant effect and/or differences between groups (see Figure 5). However, in contrast to the standard quantitative evaluation protocol, the consideration of several levels of interaction with small objects enabled us to uncover the predominant behaviors. That is, in the three strains, the most common behavioral interaction did not result in the complete burying of the marbles but their change of position (turned or partially buried).

## 4. Discussion

In the present work, we describe for the first time the in vivo effects of the PDK1 mutation in the PH domain depending on age and genetic load. Mature (11–14 months of age) male and female PDK1^−/−^ and PDK1^+/−^ PH-domain knock-in mice were studied and compared to age- and sex-matched WT mice with normal aging. The results unveil the fine-tuning of the signaling pathways by sex and genetic load, with a feminization of the behavioral profiles.

### 4.1. Homozygous-Load of the Mutant Gene Is Needed for the Reduced Somatic Growth Effects Only in Mutant Males

As previously described [33,35,36], somatic growth, measured through bodyweight, was found reduced in both male and female PDK1^−/−^ mice. It is known that homozygous male and female PDK1^−/−^ are −35% smaller from birth than WT littermates [33]. In that precedent work, magnetic resonance imaging-obtained images or physical sections of fixed organs using the Cavalieri method described reduced brain volume (−20%) but also of metabolic (liver), immune (spleen, −20%), male gonadal (testis, −50%) organs and a slight reduction in kidney size (albeit did not reach statistical significance) in PDK1^−/−^ mice compared to littermates [33]. Disector principle, a quantitative and unbiased stereological approach to estimate cell volume, also indicated that a reduction in organ volume translated into a reduction of cell size. Thus, in the case of adrenal glands of PDK1^−/−^ animals, a −40% reduction in the relative cell size of zona fasciculata cells was found compared to littermates. Interestingly, the present report shows that heterozygosis was enough to sustain normal weight. However, further analysis segregated by sex unveiled that the half genetic load of wild type PDK1 could guarantee normal somatic growth only in males since heterozygous females were already sensitive to this somatic effect of PDK1/Akt signaling pathway. We hypothesize that this sex-dependent modulation of size translates into the previously reported organ sizes, glucose resistance, hyperinsulinemia, and insulin resistance [33], and future experiments are needed to study it further.

### 4.2. Similar Neophobia and Locomotion, but Increased Anxiety-Like Phenotype in Mutant Females and Feminized Anxious-Like Phenotype of Mutant Males

In the PDK1^−/−^ mice, the cortical and hippocampal deficits in the PDK1/Akt signaling [34] and the anxious-like phenotype [36] are found attenuated at 11–14 months of age as compared to young adulthood. In the present work, we provide further evidence of a fine-tuning modulation of this signaling pathway by sex and genetic load. Neophobia, an amygdala-dependent immediate fear response to novelty, was similar among the six groups of animals. However, after that, mutant females exhibited a coping with stress strategy characterized by shorter latencies to develop the ethogram, thigmotaxis, increased wall rearing, and presence of bizarre rearings. As previously described, according to their temporal (repetitive/stereotyped or not) and spacial (horizontal/vertical) features, behaviors apparently without a purpose but considered coping-with-stress strategies can be classified as stereotyped stretching, stereotyped rearing, backward movement, and jumping [44]. These disrupted behaviors are scarcely observed in young animals and still difficult to record at middle age, as shown here by the low number in male WT. However, in C57bL/6 mice, we described bizarre behavior that could be conspicuous at 6 months of age when confronting the anxiogenic environments such as the open-field test, mainly in females due to their increased anxious-like profiles as compared to males, or when these responses are found exacerbated by neuropathological conditions [10,44,46]. Here, the bizarre emergent behavior was vertical rearing, which resembles escape behavior in the behavioral despair test. More importantly, in the present work, this female pattern was emulated by homozygous PDK1^−/−^ and heterozygous PDK1^+/−^ male mice, to the extent that the male WT profile was dissonant with the one exhibited by all the other groups. We have shown that bizarre behaviors delay the exploratory activity in adults [44] and aged mice [46], and can be modulated by early-life interventions [44]. Therefore, the selective effects of sex and PDK/Akt signaling genotype-load on the vertical but not the horizontal activity, as shown by normal habituation curves, is noticeable and suggests that underlying mechanisms are mediated by anxiety but not by hyperactivity [47,48].

### 4.3. Performance of Mutants Can Resemble Normal under Naturalistic Anxiogenic Conditions

In the precedent work [36], we demonstrated that in the PDK1 homozygous mice, anxiety but not working memory was modulated by age with a reduction in its expression at 11–13 months of age. This would also explain that here, similarly to previous work [44], the anxiogenic environment of the open and illuminated field test was found to be the best to observe the elicitation of bizarre behaviors, as well the fine-tuning of genotype and sex modulation, but when the behavior of animals was assessed in the marble burying test, behaviors did not differ. Thus, in the current work, similar signatures were shown by the different groups and the ‘moved or semi-buried marbles’ was found the dominant behavioral readout at the end of the 30 min test. Compared to an anxiogenic open-field test, marble burying is a neuroethological paradigm eliciting spontaneous responses of vigorous and deep digging of beddings to bury the pieces (food pellets or small objects) the animal finds in its environment [49,50]. The interpretation of this test is in constant debate, since it is sensitive to anxiolytic but also antipsychotic drugs [45,51], and it is proposed for modeling compulsive-like characteristics of OCD or autism spectrum disorders [51,52]. Here, as in other research, its use was aimed to in vivo identification of biological impacts in mice [53,54], in our case of genetic load and sex. Furthermore, digging can also be understood as a measure of general activity rather than a measure of repetitive or anxiety-related behavior [51,55,56,57] and conversely, the animal’s general activity can be a confounding factor. Due to this controversy, the open-field or other anxiety tests that also monitor the general activity are a must for interpretations and discard confounding factors. In all cases, in the present work, the impact of sex and genetic load on specific vertical but not on locomotor activity may also agree with the similar signatures observed in this paradigm. This would also agree with reports on drug-induced dose-dependent reduction in marble-burying independently of its locomotor effects [58] or our most recent report in a model for neuropathological aging [47].

## 5. Conclusions

Of the two unconditioned tests used, the forced (open-field) but not naturalistic (marble arena) anxiogenic environmental conditions were sensitive to detect sex and genetic-load differences at middle age in the PDK1 mutant mice. Thus, despite similar initial fear response (freezing indicating increased neophobia) and horizontal locomotion among the six groups of animals, females exhibited faster ethograms than males, with increased thigmotaxis with shorter latencies to reach the periphery and perform wall rearings, and increased wall and bizarre rearing. Genotype-load unveiled increased anxiety in males in elicitation of male ethograms and profiles resembling the performances characteristic of the female phenotype. While a heterozygous genotype-load was enough to elicit reduced somatic growth (bodyweight) in females, an homozygotic load of PDK1 was needed to exert this somatic effect in males. In summary, factor interactions indicated the complex interplay in the involvement of PI3K/Akt signaling pathway in the elicitation of different NVS’s construct items and somatic growth and the relevance of genotype-load and sex in the fine-tuning of its intensity.

## Data Availability

Not applicable.
